# Effects of medication reviews on use of potentially inappropriate medications in elderly patients; a cross-sectional study in Swedish primary care

**DOI:** 10.1186/s12913-018-3425-y

**Published:** 2018-08-07

**Authors:** Cecilia Lenander, Åsa Bondesson, Nina Viberg, Anders Beckman, Patrik Midlöv

**Affiliations:** 1 Department of Clinical Sciences in Malmö, Lund University, Jan Waldenströms gata 35, SE-205 02 Malmö, Sweden; 2 Department of Medicines Management and Informatics, Region Skåne, Sweden; 3 Department of Public Health Sciences, Karolinska Institutet, Solna, Sweden

**Keywords:** Elderly, Primary care, Medication review, Drug-related problems, Potential inappropriate medication, Clinical pharmacist

## Abstract

**Background:**

Drug use among the elderly population is generally extensive and the use of potentially inappropriate medications (PIMs) is common, which increases the risk for drug-related problems (DRP). Medication reviews are one method to improve drug therapy by identifying, preventing and solving DRPs.

The aim of this study was to evaluate the effect of medication reviews on total drug use and potentially inappropriate drug use in elderly patients, as well as describe the occurrence and types of drug-related problems.

**Method:**

This was a cross-sectional analysis to study medication reviews conducted by trained clinical pharmacists followed by team-based discussions with general practitioners (GPs) and nurses, for elderly primary care patients in Skåne, Sweden. Included in the analysis were patients ≥75 years living in nursing homes or in their own homes with home care, who received a medication review during 2011–2012. Documented DRPs were described as both the type of DRPs and as pharmacists’ recommendations to the GP. The usage of ≥3 psychotropics and PIMs (antipsychotics, anticholinergics, long-acting benzodiazepines, tramadol and propiomazine) at baseline and after medication review were also studied.

**Results:**

The analysis included a total of 1720 patients. They were on average aged 87.5 years, used typically 11.3 drugs (range 1–35) and 61% of them used 10 drugs or more. Of the patients, 84% had at least one DRP with a mean of 2.2 DRPs/patient. Of the DRPs, 12% were attributable to PIMs. The proportion of patients with ≥ one PIM was reduced significantly (*p* < 0.001) as was the use of ≥3 psychotropics (p < 0.001). The most common DRP was unnecessary drug therapy (39%), followed by dose too high (21%) and wrong drug (20%). Drug withdrawal was the most common result.

**Conclusion:**

This study shows that medication reviews performed in everyday care are one way of improving drug use among elderly patients. The use of potentially inappropriate medications and use of three or more psychotropic drugs decreased after the medication review. Our study also shows that drug use is extensive in nursing home residents and elderly patients with homecare, and that unnecessary drug therapy is a common problem.

## Background

Demographic data estimates that 22% of the global population will be older than 65 by 2050 [1]. In Sweden, the proportion of the population aged 65 years or older was 19.8% in 2015 and this is estimated to reach 23% by the year 2050 [2]. Elderly patients with multiple diseases and polypharmacy risk suffering from drug-related problems [3–5], and a substantial proportion of hospital admissions among elderly are due to adverse drug events (ADEs) [5–7]. The majority of these hospital admissions are avoidable [5, 8].

A previous study conducted in primary care by our group showed that as many as 93% of the studied elderly patients had at least one drug-related problem (DRP). Among these patients the average number of DRPs was 2.5 per patient [9]. Studies from primary care in other countries found an average of 3.5–5.5 DRPs per patient [3, 4, 10], while studies done in hospitals report 2.6–6.4 DRPs per patient [11–13]. One way of preventing and solving DRPs among the elderly is to carry out medication reviews. A medication review is a method to, in a structured and systematic way, according to local guidelines and routines, analyse, follow-up and review an individual’s drug therapy [14]. Potentially inappropriate medications (PIMs) are one cause of DRPs. In many countries, guidelines concerning potentially inappropriate medication have been developed with Beers criteria being the best-known [15, 16]. As many of the drugs listed as PIMs in Beers criteria are unavailable in Europe, development of criteria corresponding to European drug formularies have been done. These include for example the Swedish quality indicators developed by the Swedish National Board of Health and Welfare [17], the STOPP/START criteria [18] and the EU(7)-PIM list [19]. These guidelines point out inappropriate medications for the elderly on a population basis. However, there are still individuals that might need such medications. Another way of identifying inappropriate medications is the Medication Appropriateness Index (MAI) [20]; an instrument that determines a drug’s suitability to an individual, and has been validated for evaluating drug use in the elderly [21]. MAI, however, is a time-consuming instrument and less convenient to use in everyday care.

In a hospital setting, medication reviews have been reported to improve drug use [22, 23] and to reduce repeat hospital visits [11]. Medication reviews in primary care can reduce total number of drugs, reduce falls and maintain self-rated health [24, 25]. The Lund Integrated Medicine Management (LIMM) [26] is an in-hospital intervention model with multi-professional teams, including clinical pharmacists. It has been shown to reduce potentially inappropriate medications and unscheduled drug-related hospital re-visits [26]. Medication reviews have been conducted in primary care in Skåne County for over 10 years in different projects including to improve patient safety and medication use in the elderly. A study evaluating the LIMM model adapted for primary care has shown a decrease in the total number of drugs and prescription of potentially inappropriate medications for the elderly [9].

There remains a need for further analysis of the extent of elderly patients in primary care that are suffering from drug-related problems and what type of problems they present. A further need is additional data from a larger patient group on how medication reviews affect the use of potentially inappropriate medications in elderly in primary care.

The aim of this study was to evaluate the effect of medication reviews according to LIMM in elderly patients in primary care regarding total drug use and potentially inappropriate drug use, as well as describe the occurrence and types of drug-related problems present.

## Method

This study was a cross-sectional analysis to examine the process of multi-professional medication reviews on elderly primary care patients in Skåne, Sweden. It was based on everyday clinical practice in primary care with regular GPs, clinical pharmacists and nurses.

### Setting

In Sweden almost all patients are registered with a general practitioner (GP) as their primary care provider. The GP treats patients of all ages with a range of health problems, including patients in nursing homes. Skåne County, located in southernmost Sweden, has 1.3 million inhabitants with the majority of them living close to the western coast. Primary care in Skåne is funded by the county council and financed by taxes, but can be provided by both public and private primary care centres. Of the 150 primary care centres in Skåne, slightly more than 40% were private during the study period. Medication reviews guided by clinical pharmacists were in 2011–2012 offered the private primary care centres in Skåne and a total of 25 centres accepted. Involved in the medication reviews were seven clinical pharmacists, which all had at least three years’ experience of performing medication reviews. Included in this study were medication reviews performed in patients aged ≥75 years, living in nursing homes or their own homes with municipally provided home care. Only the first medication review was included in the analysis for patients receiving more than one.

### Patients

We recorded age, gender, type of housing (nursing home or in their own home with home care), number of medications and types of PIMs for all patients. Some patients (or relatives) refused to participate and if resources did not allow for all patients at a nursing home to have a medication review, the GP or nurse decided on which patients to include.

### Potentially inappropriate medications

PIMs were identified according to the Swedish National Board of Health and Welfare’s quality indicators for drug use in the elderly [17]. In these usage of ≥1 PIM, ≥3 psychotropics or ≥ 10 medications was classified as an indicator of higher risk for adverse events.

### Medication reviews according to the LIMM model

A symptom assessment scale, Pharmacotherapeutical Symptom Evaluation, 20 questions (PHASE-20) [27, 28], was used to estimate the current health status of the patient. This scale consists of medical information including present diagnosis, blood pressure, pulse, and creatinine levels, as well as questions about symptoms such as fatigue, pain, and constipation. PHASE-20 is recommended by the Swedish National Board of Health and Welfare [17] and is validated for use in connection with medication reviews for identifying possible drug-related symptoms in older people. The tool has been recommended for use in medication reviews. The nurse filled out the evaluation together with the patient. For patients unable to do this, the nurse would get assistance from the nursing assistants. This information and a copy of the medication list were sent to the pharmacist one to two weeks prior to the team-meeting.

To identify potential DRPs, the pharmacist initiated medication reviews based on the background information (symptom assessment form, including some medical information, and the medication list), but with no access to the medical record during this phase. By using forms from the LIMM model [26], the process was carried out in a structured way.

The following predetermined risk categories for identifying DRPs were used by the pharmacist to ensure structure and consistency [26]:

1. Drugs requiring therapeutic monitoring
2. Potentially inappropriate drugs for the elderly according to The Swedish National Board of Health and Welfare (PIMs)
3. Drugs that are not recommended according to the regional drug and therapeutics committee
4. Problems with administration/handling of the drug (crush, cut, inhalation technique)
5. C/D drug–drug interactions (C interactions are those involving a drug combination that could require dose adjustment; D interactions are those involving a drug combination that ought to be avoided)
6. Drug type or drug dosage not adjusted for the patient (renal or liver function)
7. Unclear indication for drug treatment
8. Suboptimal treatment
9. Drugs causing potential adverse drug reaction

After identification, the DRPs were classified by the pharmacist into seven categories of DRPs defined by Cipolle, Strand and Morley [14]:

1. Need for additional therapy
2. Unnecessary drug therapy
3. Wrong drug
4. Dose too low
5. Adverse drug reaction
6. Dose too high
7. Adherence problems

To aid the pharmacists to classify in a similar way, examples for each group were produced.

Based on the identified potential DRPs and the information sent by the nurse, the pharmacist suggested intervention recommendations. These were pre-defined and included [26]:

1. For information/notification
2. Initiation of drug therapy
3. Withdrawal of drug therapy
4. Decreased dose
5. Increased dose
6. Dose regimen adjustment
7. Change in drug formulation
8. Change of drug therapy
9. Evaluation of drug therapy

At a team meeting at the primary care centre, or at the nursing home, the identified potential DRPs and possible interventions were discussed by the patient-responsible GP, the nurse, the pharmacist and in some cases the caregiver. The pharmacist identified and selected which DRPs to discuss, but the GP and nurse was able to add to the list. At this meeting the team had access to the medical record and, if it was held at the nursing home, the nurse had access to the nursing journal. Based on these discussions and her/his clinical knowledge of the patient, the GP then decided on interventions. The changes in patient status (better, worse or unchanged) were to be followed-up by the nurse in 4–8 weeks and forwarded to the pharmacist.

For each patient the following were recorded by the pharmacist: age, gender, number of medications and type of PIM. If a prescription was for both continuous use and as needed, it was counted as one drug. Drugs for topical use such as eye drops, moisturisers and topical steroids were included; short-term antibiotic prescriptions were not. Information about DRPs discussed, suggested recommendations, agreed upon interventions and follow-up were also recorded.

### Data collection

All parts of the medication reviews, as described above, were performed by the participating pharmacists. Upon completion, the pharmacists entered all information into an Access database. In addition, the research team retained the paper records: medication lists, symptom assessments, notes on identified and discussed DRPs, on actions taken and if any follow-up was recorded.

### Data analysis

Descriptive analysis included average age and sex distribution of the patients, as well as the average number of drugs per patient. “Before medication review” indicates the treatments used prior to the of medication review being performed. “After medication review” is the result of changes in therapy decided upon during the team meeting. In accordance with the Swedish quality indicators [17] the percentage of patients taking 10 or more medications (regularly or as needed) and the percentage of patients taking three or more psychotropic drugs (from one or more of the following Anatomical Therapeutic Chemical Classification System (ATC) [29] groups; N05A (antipsychotics), N05B (anxiolytics), N05C (hypnotics and sedatives) and N06A (antidepressants)) was measured.

Occurrence of DRPs, distribution of types of DRPs, proposed intervention recommendations and the related treatment adjustments, and outcomes of follow-up were determined.

Changes in the proportion of patients taking PIMs, as defined in the Swedish quality indicators [17], and including one or more of the following drugs were analysed: antipsychotics (N05A, excluding lithium (N05AN)), drugs with anticholinergic effects (R06AD, G04 and N05BB; for example promethazine, urologic spasmolytics and hydroxyzine), long-acting benzodiazepines (N05BA01, N05CD02 and N05CD03; nitrazepam, flunitrazepam and diazepam), tramadol (N02AX) and propiomazine (N05CM).

Data were analysed using IBM SPSS version 22 [30]. We used Student’s t-test to compare groups (nursing home residents vs patients living at home with home care). Multiple significance was tested according to the suggestion by Bland, Altman [31]. Online Chi-square Calculator [32] was used to compare medication use before and after medication review as well as to compare follow-up between groups.

A level of significance of 0.05 was used.

## Results

Included in the analysis were a total of 1720 patients.

The mean age of the included patients were 87.5 years and they used on average 11.3 drugs (range 1–35). A majority of the patients were females and lived in nursing homes (Table 1.)

**Table 1:** Baseline characteristics of included patients (*n* = 1720)

	Nursing home *n* = 1508	Home care *n* = 212	*p*-value
Age, mean (SD)	87.7 (5.8)	86.3 (5.7)	< 0.01^a^
Female n (%)	1123 (74.5)	142 (67.0)	0.02^b^
Number of drugs per patient, mean (SD)	11.2 (4.6)	11.4 (4.3)	0.59^a^
continuous drugs, mean (SD)	8.5 (3.6)	9.2 (3.3)	< 0.01^a^
drugs as needed, mean (SD)	2.8 (2.1)	2.2 (2.0)	< 0.01^a^
Number of potentially inappropriate drugs, mean (SD)	0.30 (0.46)	0.27 (0.44)	0.35^a^
Antipsychotics, mean (SD)	0.12 (0.33)	0.05 (0.21)	< 0.01^a^
Long-acting benzodiazepines, mean (SD)	0.06 (0.24)	0.10 (0.31)	0.04^a^
Anticholinergics, mean (SD)	0.09 (0.29)	0.09 (0.29)	0.90^a^
Propiomazine, mean (SD)	0.02 (0.15)	0.05 (0.21)	0.09^a^
Tramadol, mean (SD)	0.05 (0.23)	0.04 (0.20)	0.49^a^
Patients with ≥1 PIM, n (%)	453 (30.0)	57 (26.9)	0.35^b^
Patients with ≥3 psychotropics, n (%)	367 (24.3)	28 (13.2)	< 0.01^b^
DRPs, mean (SD)	2.2 (1.9)	2.4 (1.8)	0.21^a^

*SD* standard deviation

^a^t-test, ^b^ Chi square test

Before the medication review, 96% of the patients used five drugs or more and 61% used 10 drugs or more. Patients with home care used more drugs for continuous use (9.2) compared to nursing home residents (8.5) (*p* = 0.004), while the latter group had more drugs for use as needed (2.8 vs 2.2, *p* < 0.001). No significant difference in total number of drugs or usage of ≥1 PIM between the groups was seen at baseline. Almost a quarter of the patients in nursing homes used ≥3 psychotropics before the medication review, which was significantly more than patients with home care (13%) (*p* < 0.001). The use of antipsychotics was more common among patients in nursing homes (12.2%) compared to patients with home care (4.7%) (*p* < 0.001), but the use of long-acting benzodiazepines was lower in nursing homes (6.0% vs 10.4%, *p* = 0.045) (Table 1).

Post medication review the mean number of drugs per patient decreased from 11.3 to 10.5.

### Drug-related problems

**Fig. 1:**
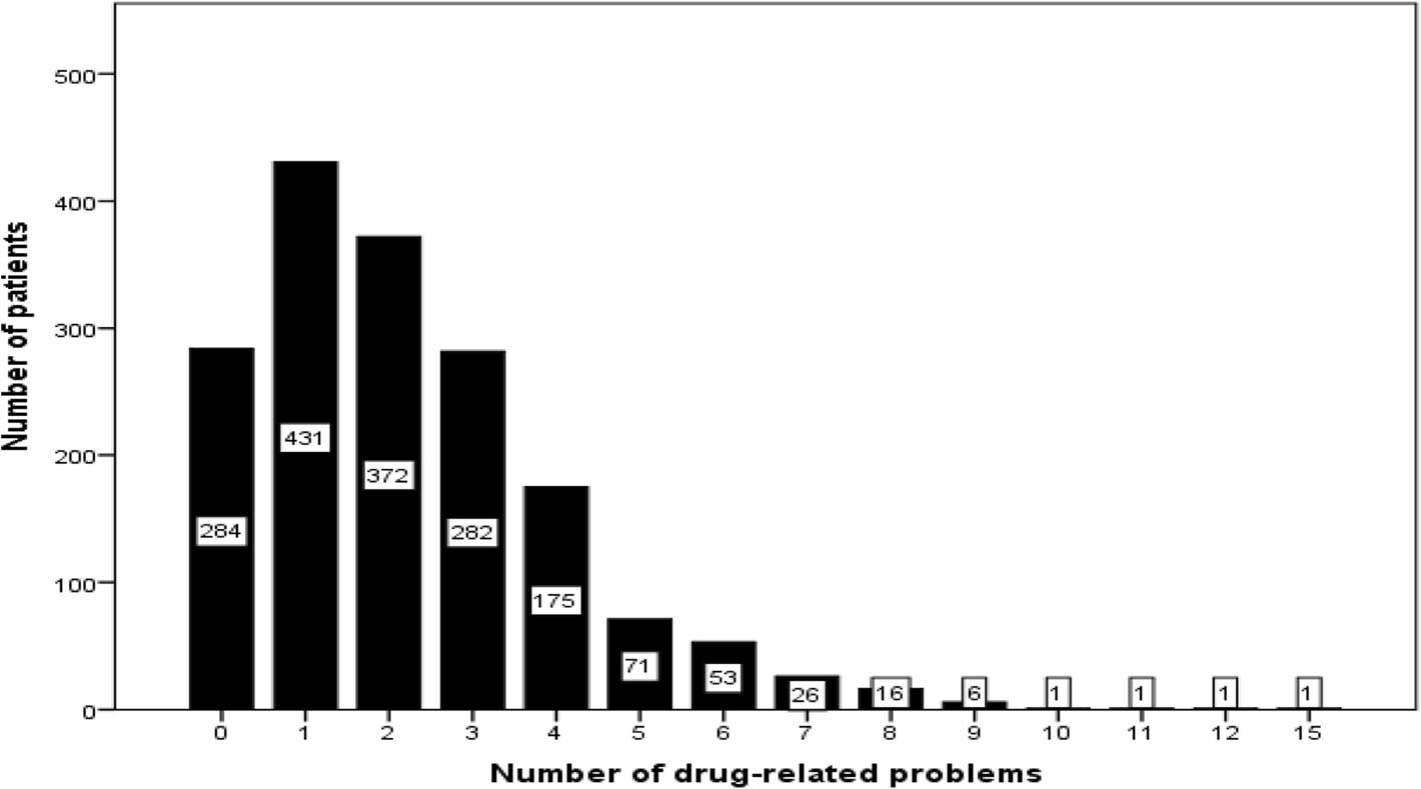
Distribution of drug-related problems (*n* = 1720 patients)

DRPs were identified in 84% (1447 of 1720) of the patients. A total of 3868 DRPs were identified and presented (range 0–15 per review), giving a mean of 2.2 DRPs per patient (Fig. 1). No significant difference was seen between the number of presented DRPs in patients at nursing homes (mean 2.2 (SD 1.9)) and patients with home care (mean 2.4 (SD 1.8)) (*p* = 0.21).

The most common drugs to cause DRPs (*n* = 3868) were those in the 1st level ATC categories “N – Nervous system” (33%), “C – Cardiovascular system” (27%) and “A – Alimentary tract and metabolism” (12%). The most common drugs to cause DRPs were low dose ASA (4.9%) followed by folic acid (4.8%), citalopram (3.6%) and simvastatin (3.3%).

Of the DRPs, 12% (485 of 3868) were attributable to PIMs.

**Fig. 2:**
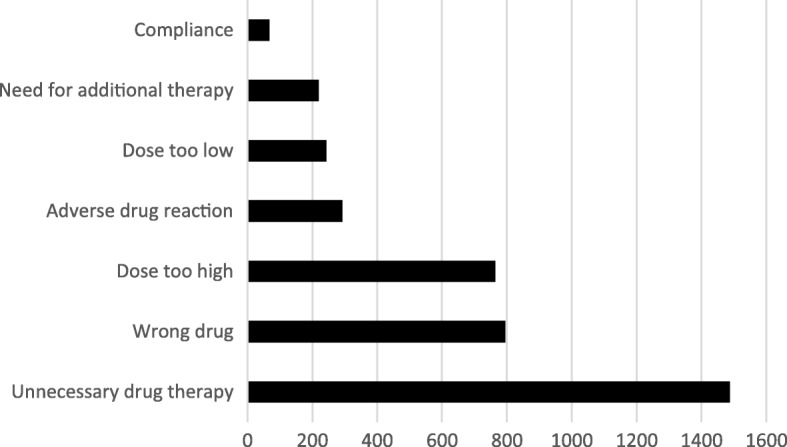
Distribution of types of drug-related problems (DRP) according to Strand, Cipolle and Morleys definition. (*n* = 3868 DRPs)

The most common types of DRPs (n = 3868) were unnecessary drug therapy (39%), dose too high (21%) and wrong drug (20%), Fig. 2.

### Suggested intervention recommendations

**Fig. 3:**
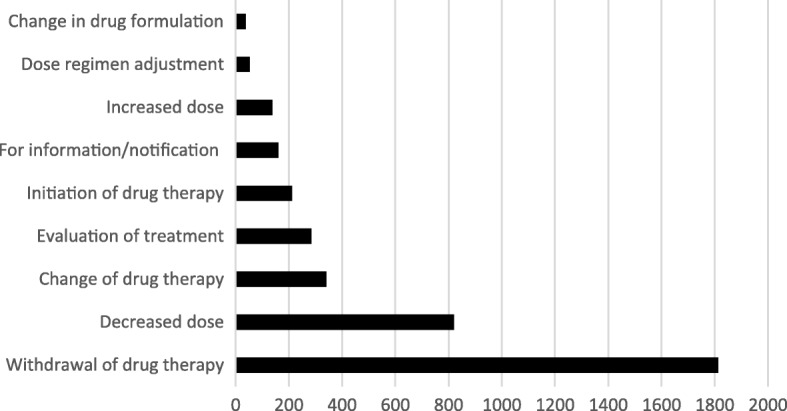
Distribution of types of suggested intervention recommendations (*n* = 3860)

Of the 3868 identified DRPs, 3860 received an intervention recommendation. The most common interventions suggested by the pharmacist to the GP were withdrawal of drug therapy (47%), decreased dose (21%) and change of drug therapy (9%) (Fig. 3). Therapy was discontinued for a total of 285 PIMs, which constituted almost 16% of the “withdrawal of drug therapy” group.

### Acceptance of suggested recommendations

For the 410 “for information/notification” recommendations acceptance or not by the GP, was not recorded. Of the remaining 3450 recommendations, the GPs accepted 80% (2760 of 3450) of the recommendations suggested by the clinical pharmacist, giving a mean of 1.60 changes per patient (range 1–15). In 9% (301 of 3450) of the cases the GP needed more information before making a decision and in 2% (77 of 3450) the GP solved the DRP in another way than suggested. In 9% (312 of 3450) of the cases the GP did not accept the pharmacists’ suggestions.

### Follow-up

For 29% (*n* = 1195) of the changes a result at follow-up was recorded. Of these, 93% (*n* = 932) led to improved (16.6%) or unchanged (76.4%) status for the patient. However, there was a significant difference between nursing homes and patients with home care. For home care patients, 31% of the changes led to an improvement in patient status compared to 15% for nursing home patients (*p* < 0.0001).

### Potentially inappropriate medications

The proportion of patients with least one PIM was reduced significantly (*p* < 0.001) (Table 2) and there was a significant decrease for all subgroups (anticholinergics, tramadol etc.). Before medication review nine patients used ≥3 PIMs and this decreased to five patients post medication review.

**Table 2:** Number of patients using potential inappropriate medications before and after medication review. (*n* = 1720 patients)

	Before medication review			After medication review			*p*-value^a^
	Total	Nursing home	Home care	Total	Nursing home	Home care	
Patients with antipsychotics, *n* (%)	194 (11.3)	184 (12.2)	10 (4.7)	136 (7.9)	128 (8.5)	8 (3.8)	< 0.001
Patients with anticholinergics, *n* (%)	158 (9.2)	139 (9.2)	19 (9.0)	72 (4.2)	60 (4.0)	12 (5.7)	< 0.001
Patients with propiomazine, *n* (%)	43 (2.5)	33 (2.2)	10 (4.7)	16 (0.9)	10 (0.7)	6 (2.8)	< 0.001
Patients with tramadol, *n* (%)	90 (5.2)	81 (5.2)	9 (4.2)	41 (2.4)	39 (2.6)	2 (0.9)	< 0.001
Patients with long-acting benzodiazepines, *n* (%)	112 (6.5)	90 (6.0)	22 (10.4)	62 (3.6)	51 (3.4)	11 (5.2)	0.001
Patients with ≥1 PIM^b^, *n* (%)	510 (29.7)	453 (30.0)	57 (26.9)	299 (17.4)	266 (17.6)	33 (15.6)	< 0.001
Patients with ≥3 psychotropics, *n* (%)	395 (23.0)	367 (24.3)	28 (13.2)	296 (17.2)	274 (18.2)	22 (10.4)	< 0.001

^b^PIM Potentially Inappropriate Medication (antipsychotics, anticholinergics, propiomazine, tramadol and long-acting benzodiazepines)

^a^Chi square test between total numbers (nursing home and home care together)

Nearly 23% (395 of 1720) of the population used ≥3 psychotropics before the medication review, and this decreased to 17% (296 of 1720) after the review (*p* < 0.001).

## Discussion

This study based in everyday clinical practice in Swedish primary care, shows that the medication reviews decreased the use of potentially inappropriate medications and the use of three or more psychotropic drugs. It also shows that drug-related problems are frequent among older patients with multi-morbidity and their most common problem is unnecessary medication.

At baseline, almost 30% of the patients in this study used at least one PIM. This is slightly higher than a Swiss study (22.5%) [33] but comparable to other Swedish studies (26–33%) [9, 34]. Compared to the general population, aged 75 years or older in the county of Skåne, use of at least one PIM was considerably higher in the study population (30% vs around 10%) indicating that the study population is less healthy than the general population [35]. The use of PIMs in the older population has decreased in recent years in Sweden [35], but the reduction is more distinct in our study. PIM usage decrease after a medication review is in line with other studies [9, 34]. There are also studies showing a correlation between usage of PIMs and falling [36].

We saw a decrease in total number of drugs and usage of PIMS. Other studies have shown that medication reviews can decrease the total number of drugs [4, 9, 25] and the use of PIMs [9] as well as reduce the incidence of falls [25]. This may in some way affect health care utilisation. However, these patients are frail and elderly and other factors, like the fact that most of them suffer from multi-morbidity, can influence their health care utilisation [37, 38].

In our study, 61% of the patients used 10 drugs or more, which is much higher than for the total 75+ population in Skåne (10.2% in 2013 [35]). In Sweden, nursing homes are usually for patients who are not able to care for themselves anymore, often because of multi-morbidity and thereby polypharmacy, which is supported by this study. With the high number of patients using 10 drugs or more, and unnecessary drug treatment being a common problem, effective ways to improve medication use in the elderly should be highly prioritised.

The population in this study is of similar age and had the same number of medications as in other studies [3, 4, 9, 39] performed in primary care. However, compared to these studies [4, 9, 39] fewer patients had at least one DRP in our study, 84% compared to 87, 93 and 98% respectively. This could be due to a larger, non-selected patient group in this study. As a result of this the mean number of DRPs in this study was lower (2.2) than in the other studies mentioned above (2.5–3.5). In this study, the most common DRP was unnecessary medication, which could be any kind of medication including PIMs. Unnecessary medication being the most common DRP is in line with other studies [3, 4], as well as medications from Anatomical Classification System classes “N- Nervous system”, “C - Cardiovascular system” and “A - Alimentary tract and metabolism”, causing the majority of DRPs. Changes in guidelines or recommendations during this time period, e.g. for folic acid and low dose ASA, may explain some of the DRPs in this study. Since almost all patients at Swedish nursing homes receive assistance with administration of drugs, compliance was not a common DRP in our study. The most common result of the medication reviews was withdrawal of drug therapy, which resulted in a decrease in both total number of drugs per patient and in PIMs.

Seven clinical pharmacists participated in the medication reviews together with many GPs and nurses. The GPs accepted 80% of the clinical pharmacists’ suggestions, which indicates that this is a feasible method to use in primary care. A study rating the clinical importance of a sample of these DRPs and suggestions showed a significant clinical importance in the vast majority of them [40]. The study also included many GPs and patients from 25 different primary care centres. These were from all over Skåne, both large and small centres, and from urban and rural areas. Identification of DRPs was done according to a well-documented method (LIMM) using both the medication list and the symptom assessment (Phase-20). Another strength is that all medication reviews were conducted as team-based discussions.

There are some limitations to this study. At the included primary care centres, not all patients received a medication review due to lack of resources (i.e. clinical pharmacists) and the medication reviews were done in everyday clinical practice. The pharmacist did not meet the patients and had no access to the patient records when preparing the medication reviews and identifying potential DRPs. At the team-meeting, the team had access to the patient records and with the GPs knowledge of the patient, the actual DRPs were clarified. Another limitation is the lack of follow-up in a majority of the medication reviews. This was supposed to be done by the nurses as a part of ordinary care. The evaluation might have been performed but forwarding the results to the pharmacist was forgotten about. As we evaluated medication reviews in everyday practice, there were no extra resources for things such as reminders to nurses. Another limitation could be the absence of a commonly used method for performing medication reviews and identifying PIMs. Since this was an evaluation of medication reviews performed according to a well-documented Swedish method (LIMM), we used the Swedish quality indicators [17] as the base for our analysis. These indicators are well-known and generally well accepted among Swedish GPs. To avoid mass significance, we checked, by multiplying the observed *p*-values with the number of tests, that our results where significant at the 0.05 level according to Bland, Altman [31].

Further studies, like a larger randomised controlled study comparing different settings, are needed, especially to see possible effects of medication reviews on health care utilisation.

## Conclusion

This study shows that medication reviews performed in everyday care are one way of improving drug use among elderly patients. The use of potentially inappropriate medications and use of three or more psychotropic drugs decreased after a medication review. The study also shows that drug use is extensive in nursing home residents and elderly patients with homecare, and unnecessary drug therapy is a common problem in this population.

## Abbriviations

ADE: Adverse Drug Event.
ATC: Anatomical Therapeutic Chemical Classification System.
DRP: Drug-Related Problem.
GP: General Practitioner.
LIMM: Lund Integrated Medicines Management.
MAI: Medication Appropriateness Index.
PHASE-20: Pharmacotherapeutical Symptom Evaluation, 20 questions.
PIM: Potentially Inappropriate Medication.
